# Characteristics and Short-Term Surgical Outcomes of Patients with Recurrent Lumbar Disc Herniation after Percutaneous Laser Disc Decompression

**DOI:** 10.3390/medicina57111225

**Published:** 2021-11-10

**Authors:** Hidetomi Terai, Koji Tamai, Masayoshi Iwamae, Kunikazu Kaneda, Hiroshi Katsuda, Nagakazu Shimada, Hiroaki Nakamura

**Affiliations:** 1 Department of Orthopaedic Surgery, Graduate School of Medicine, Osaka City University, Osaka 545-8585, Japan; hterai@med.osaka-cu.ac.jp (H.T.); iwamae.masayoshi@med.osaka-cu.ac.jp (M.I.); hnakamura@med.osaka-cu.ac.jp (H.N.); 2 Department of Orthopaedic Surgery, Shimada Hospital, Habikino City, Osaka 583-0875, Japan; kaneda@heartful-health.or.jp (K.K.); hkatsuda@heartful-health.or.jp (H.K.); nagakazu_shimada@heartful-health.or.jp (N.S.)

**Keywords:** recurrence, lumbar disc herniation, percutaneous laser disc decompression, microendoscopic decompression, outcomes

## Abstract

*Background and Objectives*: Although percutaneous laser disc decompression (PLDD) is one of the common treatment methods for patients with lumbar disc herniation (LDH), the recurrence of LDH after PLDD is estimated at 4–5%. This study compares the preoperative clinical data and clinical outcomes of patients who underwent primary microendoscopic discectomy (MED) or MED following PLDD. *Materials and Methods*: We retrospectively analyzed 2678 patients who underwent MED for LDH. The PLDD group included patients with previous PLDD history at the same level of LDH, and a matched control group was created using propensity score matching for age, sex, and body mass index. Preoperative data, preoperative radiographic findings, and surgical data of the groups were compared. To compare postoperative changes in clinical scores between the groups, a mixed-effect model was used. *Results*: As a result, 42 patients (1.6%) had previously undergone PLDD, and a control group with 42 patients were created. The disc degeneration severity was not significantly different between the groups. However, Modic changes were more frequent in the PLDD group than in the matched control group (*p* = 0.028). There were no significant differences in dural adhesion rate or surgery-related complications including dural injury, length of stay, and recurrence rate of LDH after surgery. In addition, the improvement of clinical scores did not significantly differ between the two groups (*p* = 0.112, 0.913, respectively). *Conclusions*: We concluded that patients with recurrent LDH after PLDD have advanced endplate degeneration, which may reflect endplate injury from a previous PLDD. However, a previous history of PLDD does not have a negative impact on the clinical result of MED.

## 1. Introduction

Lumbar disc herniation (LDH) is the most common cause of lumbosacral radicular syndrome and low back pain and affects 1–2% of the general population in the United States annually [1], placing a significant burden on healthcare services and the economy worldwide [2,3]. Surgical intervention is recommended for patients with LDH who are non-responsive to at least 6 weeks of non-surgical treatment [4]. Previously, open discectomy was used to treat patients with LDH; however, the incidence of low back pain following open discectomy surgery is almost 30% [5], and rates of revision surgery of up to 20% have been reported [6]. To overcome such disadvantages, minimally invasive decompression procedures including percutaneous laser disc decompression (PLDD) techniques have been developed [7].

Although PLDD allows physicians to decrease complications after surgery [8], the recurrence of LDH remains as a serious complication. The recurrence rate of LDH after PLDD is estimated at 4–5% [9]. It is well established that the clinical outcomes of reoperations for patients with recurrent LDH after open surgery are inferior to those of the primary surgery, with a higher incidence of complications after reoperations [10]. However, there is no report that evaluates the surgical outcomes of recurrent LDH after PLDD. We hypothesize that the surgical outcomes of recurrent LDH after PLDD are inferior to the outcomes of primary surgery due to nerve root and dural adhesion reflecting the inflammatory reaction of PLDD.

The microendoscopic discectomy (MED) technique is a current standard treatment method for patients with LDH, and MED results in less postoperative pain and a quicker return to work compared to open surgery [11]. Based on our institution’s surgical strategy, all patients with LDH are treated with MED procedures regardless of a previous history of PLDD. Therefore, our database of patients with LDH enabled us to test our hypothesis.

The primary aim of the current study was to compare the clinical outcomes of patients subjected to primary MED and MED following PLDD using propensity score matching. Additionally, we compared the preoperative clinical data of the two groups to identify characteristics of LDH after PLDD.

## 2. Materials and Methods

### 2.1. Ethics Approval

We conducted a retrospective cohort study. All study participants provided informed consent, and the study protocol was approved by the Institutional Review Board of Shimada Hospital.

### 2.2. Study Population

We reviewed medical records of 4829 consecutive patients who underwent microendoscopic posterior decompression surgery at Shimada Hospital between 2005 and 2019 and were followed up for at least 3 months postoperatively (1692 females, 3137 males; mean age at surgery, 56.3 ± 17.7 years). The diagnoses included lumbar disc herniation (*n* = 2243), lumbar spinal stenosis (*n* = 1548), lumbar spinal stenosis with disc herniation (*n* = 435), foraminal stenosis (*n* = 131), and others such as facet cyst and far-out syndrome (*n* = 513). In total, 2678 patients with disc herniation treated with discectomy were enrolled in our study.

### 2.3. Surgical Criteria and Preoperative Care

Posterior endoscopic discectomy was indicated in patients who had neurogenic claudication or radicular pain with associated neurological signs, disc herniation on 1.5 T magnetic resonance imaging (MRI) at an intervertebral level that explained their symptoms and did not improve despite adequate conservative treatment of at least three months. Exclusion criteria were as follows: spondylolisthesis > grade 2, spondylolytic–spondylolisthesis, and degenerative lumbar scoliosis with a Cobb angle > 20°. All patients were treated according to the standardized care pathways of our institution. The MED procedure was performed under general anesthesia as previously reported [12]. All patients were allowed to sit and walk on the day after surgery. The care pathway included the routine use of celecoxib (200 mg per day for 7 days) for pain after surgery and allowed the use of additional painkillers (acetaminophen, opioids, or non-steroidal anti-inflammatory drugs) as needed. All patients underwent lumbar MRI three days postoperatively and were recommended for hospital discharge 6 or 7 days postoperatively.

### 2.4. Collective Data

#### 2.4.1. Preoperative Data

Data regarding the patients’ age at surgery, sex, height, weight, body mass index (BMI), history of preoperative PLDD at the same surgical level, comorbidities (diabetes mellitus, hypertension, cardiac diseases, respiratory diseases, and cerebrovascular diseases), and preoperative symptoms including motor weakness and bowel bladder dysfunction were collected from the medical records.

#### 2.4.2. Radiologic Data

Preoperative MRIs were used to determine the severity of disc degeneration, endplate degeneration, and types of herniation. The severities of disc degeneration of the surgical level were evaluated using Pfirrmann classifications [13]. The results were summarized according to two levels of severity: no/mild degeneration including grades 1, 2, and 3; and severe degeneration including grades 4 and 5. Changes of endplate and sub-chondral bone at the surgical level were evaluated using Modic classification [14]. The types of herniation were classified into three patterns: subligamentous extrusion, transligamentous extrusion, and sequestration.

#### 2.4.3. Surgical Data

Surgical data, including the total operative time and findings during surgery regarding nerve root adhesion, dural puncture, and the existence of a hard disc, were collected. Postoperative surgical data, including neurological deterioration, length of stay, the use of additional painkillers, the presence of hematoma on MRI, and the recurrence of LDH at the same level were collected. The use of additional painkillers was defined as the use of a painkiller in addition to the routinely prescribed celecoxib. The number of times of use was recorded as additional painkiller use. Postoperative hematoma was assessed via MRI three days postoperatively and defined as dura compressed by a hematoma at the level of the decompression surgery. When a patient underwent reoperation for recurrent LDH after MED, the case was recorded as recurrence of same-level LDH.

#### 2.4.4. Clinical Scores

Japanese Orthopedic Association (JOA) scores for degenerative lumbar disease and Oswestry Disability Indexes (ODI) were collected preoperatively and at 3 months postoperatively [15,16]. The JOA score is a scoring system that evaluates the severity of LDH symptoms and is assessed and recorded by a physician (29 points: best, 0 points: worst). ODI is a patient-reported assessment evaluating outcomes specific to lumbar disease via 10 segments (0 points: best, 100 points: worst).

### 2.5. Statistical Analysis

All patients were first classified into two groups based on their past history: a PLDD group and a control group. The average age, average preoperative clinical score, ratio of males to females, and BMI were compared between these two groups using the chi-squared test for categorical variables and the Mann–Whitney U test for continuous variables. Subsequently, matched control groups were created using propensity score matching. To estimate the propensity score, we fitted a logistic regression model using the patient’s age, sex, and BMI. The nearest-neighbor matching procedure was used, with the restriction that the matched propensities had to be within 0.001 units of each other. To identify the characteristics associated with the patients who underwent PLDD, preoperative data, preoperative radiographic findings, and surgical data were compared between the PLDD group and the matched control group using the Mann–Whitney U test or chi-squared test. When there was a significant difference in the chi-squared test, the residual analysis was performed as post-hoc analysis. The result of the residual analysis was considered to indicate *p* < 0.05 when all variables had |r| > 1.96, in accordance with the Haberman method [17]. To compare the postoperative changes in the JOA score and ODI between the PLDD group and the matched control group, a mixed-effect model was applied. All analyses were performed using SPSS software (version 23; SPSS, Chicago, IL, USA). A value of *p* < 0.05 was considered statistically significant.

## 3. Results

### 3.1. Overall Comparisons

**Figure 1:**
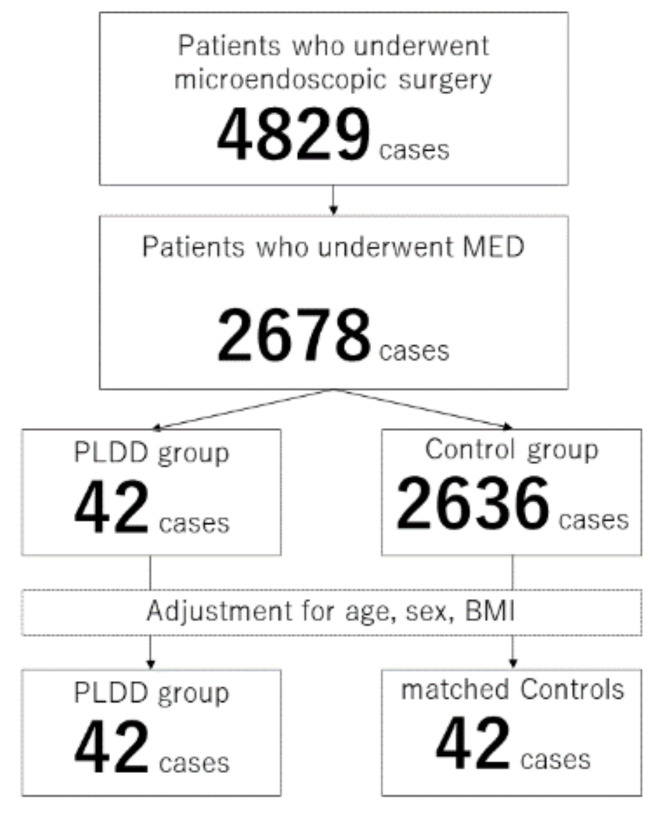
Flowchart of the current study.

Among 2678 patients with disc herniation treated with discectomy, 42 patients (1.6%) had previously undergone PLDD at the surgical level (Figure 1). The average period between the previous PLDD and MED was 36.7 ± 55.9 months and ranged from 3 weeks to 18 years. There were significant differences in the sex ratio (*p* = 0.005), body weight (*p* = 0.007), and body height (*p* = 0.024) between patients who had undergone PLDD (*n* = 42) and those who had not (*n* = 2636) (Table 1).

### 3.2. Comparisons between the PLDD Group and the Matched Control Group

After adjusting for age, sex, and BMI, the preoperative data, surgical data, and improvement of clinical scores of the PLDD group (*n* = 42) and matched controls (*n* = 42) were compared (Figure 1).

### 3.3. Preoperative Data

In addition to the age, sex, and BMI, there were no significant differences in comorbidities or preoperative symptoms including JOA scores, ODI scores, and motor weaknesses between the PLDD group and the matched control group (Table 2).

### 3.4. Radiographic Findings

There was no significant difference in the severity of disc degeneration between the PLDD group and the matched controls (Table 3). However, the Modic changes were more frequent in the PLDD group than in the matched control group (*p* = 0.028). One case of Modic type 1, 26 cases of type 2, and 1 case of type 3 were found in the PLDD group, while the matched control group included zero cases of Modic type 1, 15 cases of type 2, and 2 cases of type 3. The herniation patterns were significantly different between the two groups (*p* = 0.011). Post-hoc residual analysis demonstrated that the PLDD group included significantly more cases with subligamentous extrusion, while the matched control group included significantly more cases with sequestration (*p* < 0.05, respectively)

### 3.5. Surgical Data

There were no significant differences in surgical time, neurological deterioration, number of hematomas, length of stay, or additional painkiller use between the two groups (Table 4). The number of patients with nerve root adhesion and dural injury was not significantly different between the two groups. However, the frequency of hard disc herniation was significantly higher in the PLDD group than in the matched control group (*p* = 0.026). There was no significant difference in LDH recurrence at the same level after surgery between the two groups (*p* = 1.000). One patient in the PLDD group had a reoperation 4 years after the MED surgery, and two patients in the matched control group had reoperations 3 months and 3 years after the primary MED.

### 3.6. Postoperative Clinical Score

**Figure 2:**
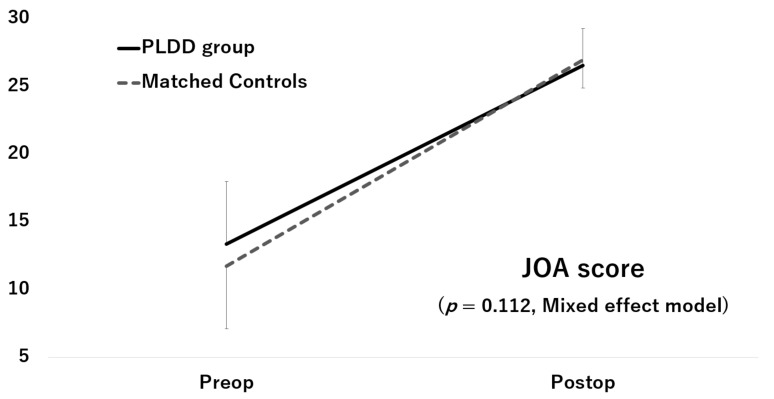
Improvement of the JOA score.

**Figure 3:**
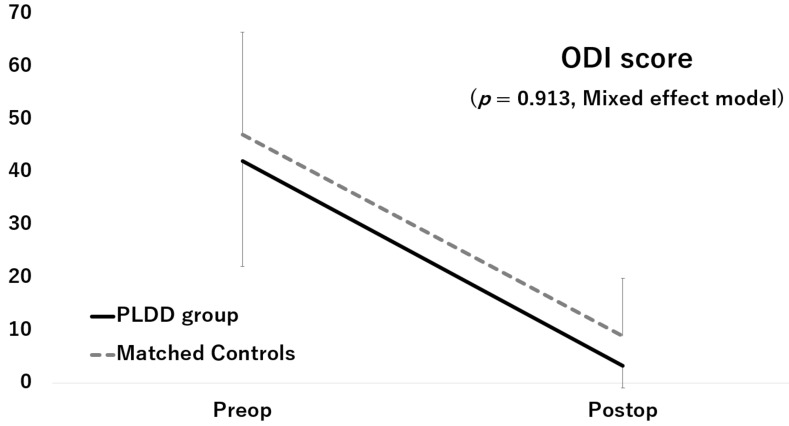
Improvement of the ODI score.

Although the JOA score and ODI score significantly improved after surgery in both groups, the improvements did not significantly differ between the two groups (JOA score: *p* = 0.112, Figure 2; ODI score: *p* = 0.913, Figure 3)

## 4. Discussion

We found that endplate degeneration, hard disc herniation, and subligamentous extrusion are more common in patients with LDH who previously underwent PLDD than in those who did not. However, contrary to our hypothesis, a previous history of PLDD does not have a negative impact on the clinical results of MED in terms of surgery-related complications, length of stay, recurrence of LDH, or short-term improvement of the clinical scores.

There were significant differences in gender and body size (height and weight) between patients with and without a history of PLDD. These differences may represent a selection bias of who receives PLDD in Japan. As the Japanese National Health Insurance system does not cover PLDD, the patients themselves must pay PLDD in full. Therefore, we applied propensity score matching calculated with the variables with significant differences in the overall comparison to minimize the effect of selection bias on the results of this study [18].

This study revealed that patients with recurrent LDH after PLDD more frequently had subligamentous herniation compared to the matched control group. This may be due to segmented nucleus pulposus resulting from previous PLDD procedures [19]. The incidence to penetrate the posterior longitudinal ligament as a lump could be lower in segmented nucleus pulposus than in the non-segmented one. Patients with recurrent LDH after PLDD also had a higher rate of endplate signal change on MRI, as well as a higher incidence of hard disc. These results support our hypothesis that previous PLDD procedures affected not only the nucleus pulposus, but also the endplate and subchondral bone, resulting in a signal change on MRI. In addition, endplate injuries may result in the detachment of the ring apophysis, which is identified as hard disc herniation during subsequent surgeries.

Contrary to our hypothesis, the surgical outcomes of patients with recurrent LDH after PLDD were not inferior to those of patients with LDH and no history of PLDD. Although peridural fibrosis formation after open surgery is a severe problem [20,21], there was no significant difference in the incidence of dural and nerve root adhesion between patients who have previously undergone PLDD and those who have not in this study. In addition, patients with recurrent LDH after PLDD maintain anatomical structures such as the laminar bone and the ligamentum flavum, as opposed to patients who undergo open surgery. These structures can be used as guideposts and help ensure a safe surgery, especially in minimally invasive surgeries including MED [22]. This study found that a history of PLDD does not have negative effects on subsequent MED procedures.

As we do not perform PLDD at our institution, we cannot evaluate the effectiveness or failure rate of PLDD. In addition, the current study cannot evaluate the adequate indication of PLDD. However, our results support the use of MED surgery for patients with recurrent LDH after PLDD. We recommend the use of wide posterior decompression when performing MED surgery in patients with LDH after PLDD, as these patients are more likely to have hard disc herniation, including the detachment of the ring apophysis.

There are several limitations to our study. Although the recurrence of LDH after MED was evaluated for an average of 65.1 months after surgery, the patients’ clinical scores were assessed three months postoperatively. A longer follow-up of the clinical scores is necessary. In addition, some elements of our standard postoperative care (for example, the length of stay) are not practical under the insurance systems in several countries. Finally, although we used propensity score matching, the retrospective nature of this study renders it difficult to exclude some types of biases. To overcome such limitations, large-scale prospective studies with long-term follow ups of patients with a previous history of PLDD should be designed with an adequate number of patients. However, this study also has several strengths. All of the cases were treated at one institution, which eliminates variations in surgical indications, surgical methods, surgical skills, and pre- and postoperative care, and detailed perioperative data were available. In addition, the number of patients in this study is the largest reported to date. Therefore, in spite of the abovementioned limitations, we believe that the results of the current study provide beneficial knowledge and aid physicians in developing an effective and safe surgical strategy for the treatment of LDH in patients with a history of PLDD.

## 5. Conclusions

Patients with recurrent LDH after PLDD are more likely to have advanced endplate degeneration and hard disc herniation, which may reflect endplate and subchondral bone injury that occurs in PLDD. However, a history of PLDD does not have a significant impact on the clinical results of subsequent MED, including surgical complications, length of stay, recurrence of LDH, and improvement of short-term clinical scores. These results should encourage spinal surgeons to perform MED surgery on patients with recurrent LDH after PLDD.

## Figures and Tables

**Table 1:** Overall comparisons.

	Overall Patients	PLDD Group	Control Group	*p* Value
Number	2578	42 (1.6%)	2636 (98.4%)	
Age (years)	48.2 ± 16.9	45.4 ± 14.9	48.3 ± 16.9	0.203 ^#^
Sex (Female/Male)	913/1765	6/36	907/1729	0.005 ^†^
Body weight (kg)	66.1 ± 13.2	70.9 ± 11.8	66.0 ± 13.3	0.007 ^#^
Body height (cm)	166.3 ± 9.3	169.5 ± 8.6	166.3 ± 9.3	0.024 ^#^
BMI (kg/m^2^)	23.8 ± 3.7	24.6 ± 2.9	23.8 ± 3.8	0.050 ^#^
Preop JOA score	12.6 ± 5.3	13.3 ± 4.6	12.6 ± 5.4	0.353 ^#^
Preop ODI score	47.9 ± 20.5	42.4 ± 20.1	48.0 ± 20.6	0.123 ^#^

*p*-value refers to the comparison between the severe and the no/mild numbness group. ^#^: Mann–Whitney U test, ^†^: Chi-squared test. PLDD: percutaneous laser disc decompression, BMI: body mass index, JOA: Japanese Orthopedic Association, ODI: Oswestry Disability Index.

**Table 2:** Comparisons of preoperative data between the PLDD group and the matched controls.

	PLDD Group	Matched Controls	*p* Value
Number	42	42	
Age (years old)	45.4 ± 14.9	46.9 ± 17.1	0.664 ^#^
Sex (Female/Male)	6/36	4/38	0.738 ^†^
Body height (cm)	169.4 ± 8.6	168.4 ± 7.4	0.534 ^#^
Body weight (kg)	70.9 ± 11.8	66.7 ± 9.9	0.098 ^#^
BMI (kg/m^2^)	24.7 ± 3.0	23.6 ± 3.3	0.144 ^#^
Comorbidity			
Diabetes mellitus	1 (2.4%)	2 (4.8%)	1.000 ^†^
Hypertension	4 (9.5%)	5 (11.9%)	1.000 ^†^
Cardiac disease	2 (4.8%)	4 (9.5%)	1.000 ^†^
Respiratory disease	1 (2.4%)	3 (7.1%)	0.616 ^†^
Cerebrovascular disorders	0	0	1.000 ^†^
Preoperative symptoms			
Motor weakness (MMT ≤ 4)	7 (16.7%)	9 (21.4%)	0.782 ^†^
Bowel bladder disfunction	0	9 (21.4%)	1.000 ^†^
JOA score	13.3 ± 4.6	11.6 ± 4.6	0.110 ^#^
ODI score	42.4 ± 20.1	47.2 ± 19.7	0.290 ^#^

^#^: Mann–Whitney U test, ^†^: Chi-squared test. PLDD: percutaneous laser disc decompression, BMI: body mass index, MMT; manual motor test, JOA: Japanese Orthopedic Association, ODI: Oswestry Disability Index.

**Table 3:** Comparisons of radiographic analysis between the PLDD group and matched controls.

	PLDD Group	Matched Controls	*p* Value
Pfirrmann classification			0.157 ^†^
No/mild degeneration (Grade 1, 2, or 3)	8 (19.0%)	13 (31.0%)	
Severe degeneration (Grade 4 or 5)	34 (81.0%)	29 (69.0%)	
Modic change			0.028 ^†^
Negative	14 (33.3%)	25 (59.5%)	
Positive (Type 1, 2, or 3)	28 (66.7%)	17 (40.5%)	
Herniation pattern			0.016 ^†^
Subligamentous extrusion	17 (40.5%)	7 (16.7%)	>0.05 *
Transligamentous extrusion	21 (50.0%)	23 (54.8%)	
Sequestration	4 (9.5%)	12 (28.6%)	>0.05 *

^†^: Chi-squared test, *: *p* < 0.05 in the residual analysis. PLDD: percutaneous laser disc decompression.

**Table 4:** Comparisons of surgical data between the PLDD group and matched controls.

	PLDD Group	Matched Controls	*p* Value
Total surgical time (min)	69.2 ± 31.4	67.3 ± 39.1	0.866 ^#^
Findings during surgery			
Nerve root adhesion	7 (16.7%)	3 (7.1%)	0.156 ^†^
Dural injury	2 (4.8%)	1 (2.4%)	1.000 ^†^
Hard disc herniation	6 (14.3%)	0	0.026 ^†^
Neurological deterioration	0	0	1.000 ^†^
In hospital period (days)	6.2 ± 1.8	6.1 ± 1.2	0.897 ^#^
Additional pain killer use	1.0 ± 0.9	0.9 ± 1.0	0.649 ^#^
Hematoma on postoperative MRI	2 (4.8%)	3 (7.1%)	1.000 ^†^
Recurrence of same level LDH	1 (2.4%)	2 (4.8%)	1.000 ^†^

PLDD: percutaneous laser disc decompression, MRI: magnetic resonance imaging. ^#^: Mann–Whitney U test, ^†^: Chi-squared test.

## Data Availability

The data presented in this study are available on request from the corresponding author.
